# Systemic Immune and miRNA Signatures Associated with Long-Term Ranibizumab Response in Neovascular Age-Related Macular Degeneration

**DOI:** 10.3390/ph19060955

**Published:** 2026-06-19

**Authors:** Laura García-Quintanilla, Pablo Almuiña-Varela, María José Rodríguez-Cid, María Gil-Martinez, Maximino J. Abraldes, Francisco Gomez-Ulla, Miguel González-Barcia, Diana Carolina Castro-Fernández, Antonio Cañizo-Outeiriño, Andrea Cuartero-Martínez, Ana Estany-Gestal, Francisco J. Otero-Espinar, Maribel Fernández-Rodríguez, Anxo Fernández-Ferreiro

**Affiliations:** 1 Hospital Pharmacy Department, University Clinical Hospital of Santiago de Compostela (SERGAS), 15706 Santiago de Compostela, Spain; lauragarqu@gmail.com (L.G.-Q.); miguel.gonzalez.barcia@sergas.es (M.G.-B.); antonio.canizo@gmail.com (A.C.-O.); 2 FarmaCHUSLab Group, Health Research Institute of Santiago de Compostela (IDIS), 15706 Santiago de Compostela, Spain; pablo.almuina@gmail.com (P.A.-V.); dianacarolinacastrofernandez@gmail.com (D.C.C.-F.); andrea.cuartero.martinez@sergas.es (A.C.-M.); 3 Ophthalmology Department, University Clinical Hospital of Santiago de Compostela (SERGAS), 15706 Santiago de Compostela, Spain; maria.jose.rodriguez.cid@sergas.es (M.J.R.-C.); mariagilmtez@hotmail.com (M.G.-M.); maxiabraldes@gmail.com (M.J.A.); 4 Instituto Oftalmológico Gómez-Ulla, 15706 Santiago de Compostela, Spain; fgomezulla@gmail.com; 5 FIDIS-Unidad de Epidemiología e Investigación Clínica, 15706 Santiago de Compostela, Spain; ana.estany.gestal@sergas.es; 6 Department of Pharmacology, Pharmacy and Pharmaceutical Technology, Faculty of Pharmacy, University of Santiago de Compostela (USC), 15705 Santiago de Compostela, Spain; francisco.otero@usc.es; 7 Paraquasil Group, Health Research Institute of Santiago de Compostela (IDIS), University Hospital Santiago de Compostela, 15706 Santiago de Compostela, Spain; 8 Institute of Materials (iMATUS), University of Santiago de Compostela (USC), University Hospital Santiago de Compostela, 15705 Santiago de Compostela, Spain

**Keywords:** ranibizumab, inflammatory, age-related macular degeneration

## Abstract

**Objectives**: To characterize the one-year functional, anatomical, and molecular responses to intravitreal Ranibizumab in treatment-naïve patients with neovascular age-related macular degeneration (nAMD), and to identify systemic immune and miRNA signatures associated with treatment response. **Methods**: This prospective longitudinal observational study included 44 treatment-naïve patients with nAMD. Patients received up to four monthly intravitreal Ranibizumab injections, followed by a treat-and-extend regimen. Best-corrected visual acuity using ETDRS letters, central retinal thickness by optical coherence tomography, fluorescein angiography, and OCT angiography were assessed at baseline and 12 months. Peripheral blood samples were collected at both time points to quantify seven circulating cytokines using an IMMULITE chemiluminescent immunoassay and to profile 37 candidate miRNAs by TaqMan OpenArray RT-qPCR from leukocyte-derived RNA. Treatment response was classified using composite anatomical and functional criteria, including intraretinal/subretinal fluid resolution, ≥25% central retinal thickness reduction, and a ≥5 ETDRS letter gain. **Results**: At one year, patients showed significant central retinal thickness reduction and overall visual stabilization, although good and poor responders differed according to composite response criteria. Statin use was numerically more frequent among poor responders, although this difference was not statistically significant. Soluble IL-2R increased significantly over time in the overall cohort, mainly driven by good responders who showed higher median levels at both visits. IL-8 also increased globally, without significant between-group differences. Among differentially expressed miRNAs, miR-3121 was the only candidate reaching statistical significance and was downregulated in good responders. ROC analysis showed moderate discriminative performance for miR-3121, with an AUC of 0.76. **Conclusions**: One-year response to Ranibizumab in nAMD may involve systemic immune activation and miRNA regulation. miR-3121 emerges as a candidate biomarker of treatment response, supporting further validation in larger independent cohorts.

## 1. Introduction

Age-related macular degeneration (AMD) is a common degenerative disease of the retina and a leading cause of irreversible blindness worldwide. Age is the major risk factor for developing AMD, and it is generally accepted that its prevalence is particularly high and increases after 55 years of age. It is estimated that more than 200 million people worldwide suffer from AMD, a number projected to rise as the global population ages, thereby positioning AMD as an important public health concern [1]. Furthermore, as a degenerative condition with a prolonged clinical course, AMD progressively impacts the health and quality of life of patients, being increasingly incapacitating and costly [2]. Pathologically, AMD is characterized by the progressive degeneration of the macula, a specialized region responsible for central vision. This degenerative process involves photoreceptor (PR) loss, retinal pigment epithelium (RPE) dysfunction, Bruch’s membrane (BrM) thickening, and choriocapillaris abnormalities. Advanced AMD can lead to irreversible vision loss through two distinct advanced forms: dry AMD and wet or neovascular AMD (nAMD). While nAMD accounts for only 10–15% of AMD cases, it is responsible for the vast majority of AMD-related blindness. nAMD is characterized by choroidal neovascularization (CNV), which often results in blood leakage, hemorrhages, and fibrosis [3].

Although the pathogenesis of AMD is complex and obscured by heterogeneity between patients and the gradual transition from physiological aging, several biological processes have been identified as drivers of the disease, including cellular senescence, impaired proteostasis, metabolic imbalance, oxidative stress, angiogenesis, inflammation, and activation of the complement cascade [4,5]. Among these mechanisms, the interplay between inflammation and angiogenesis is particularly crucial. The inflammatory hypothesis of AMD suggests that inflammation is initially an adaptive response to remove accumulated extracellular debris in the retina but becomes chronic during aging (“para-inflammation”) and eventually dysregulated, leading to tissue damage [6]. Concurrently, the accumulation of material in the subepithelial space and thickening of the BrM create a physical barrier that reduces oxygen diffusion from the choroid to retinal cells. The consequent pro-inflammatory and hypoxic environment activates signaling cascades that exacerbate inflammation through pro-inflammatory cytokines (e.g., interleukins) and vessel growth through pro-angiogenic genes, such as those encoding vascular endothelial growth factor (VEGF), which is the primary effector of pathological CNV [7]. This signaling cascade is modulated by microRNAs (miRNAs), which post-transcriptionally regulate transcription factors and protein-encoding messenger RNAs (mRNAs) [8].

Given the central role of VEGF in CNV, therapies targeting this molecule have become the standard of care for nAMD [2]. Five anti-VEGF drugs are approved and globally commercialized: Ranibizumab, Aflibercept, Brolucizumab, Faricimab, and Bevacizumab [9]. Among them, Ranibizumab has been in use for more than a decade after showing robust efficacy to stop CNV and improve visual function in pivotal clinical trials. It is a humanized monoclonal antibody antigen-binding fragment (Fab) designed to bind and inhibit all the isoforms of member A of the VEGF family (VEGF-A) [10]. Due to its smaller size compared to full-length antibodies, Ranibizumab can more easily penetrate the retina and reduce vascular growth and CNV, although it requires monthly injections [11]. In addition, while the anatomical and functional effects of Ranibizumab are well-known, its long-term molecular effects, particularly those regulating the ocular and systemic inflammatory profile, remain less characterized [12]. Understanding whether long-term inhibition of VEGF correlates with stabilization of the underlying inflammatory cascade is essential for optimizing patient management.

In this context, the present study aims to characterize the long-term functional, anatomical, and molecular responses of patients with nAMD to treatment with Ranibizumab after one year. Specifically, clinical parameters were acquired, and the expression of miRNA markers and the profile of interleukins (ILs) were assessed in plasma, both at baseline and after one year of treatment. This longitudinal analysis seeks not only to elucidate the molecular dynamics associated with long-term Ranibizumab therapy but also to identify potential molecular biomarkers capable of predicting the response to treatment. Importantly, this study addresses the systemic immuno-inflammatory and miRNA correlates of treatment response, complementing rather than substituting the local ocular processes that drive nAMD pathology.

## 2. Results

### 2.1. Cohort Description and Clinical Parameters

A total of 44 treatment-naïve patients with nAMD were enrolled, of whom 43.2% were good responders and 56.8% were poor responders after one year of Ranibizumab therapy. The median age of participants was 80 years (IQR: 74–86 years), with a predominance of females (59.1%). No significant differences in age or sex distribution were observed between good responders (*n* = 19) and poor responders (*n* = 25). Comorbid conditions were common, with 38.6% of the population classified as obese (BMI > 30 kg/m^2^) and 31.8% reporting a history of smoking; these variables were similarly distributed across response groups. The use of concomitant medications was also frequent. Anti-inflammatory drugs were taken by 29.5% of participants, while 20.5% were on anticoagulant therapy, with no meaningful differences between groups. Of particular interest, nearly half of the cohort (47.7%) was receiving statin therapy at study entry. Statin (lipid-lowering) use was more frequent among poor responders (15/25, 60.0%) than good responders (6/19, 31.6%), although this difference did not reach statistical significance (Fisher’s exact test, *p* = 0.076; OR = 3.25). Overall, demographic factors, comorbidities, and most concomitant medications were balanced between groups, with statin therapy emerging as the only baseline characteristic showing a non-significant numerical imbalance between groups. The demographic parameters of the study population in the long-term analysis are summarized in Table 1.

All patients received the same standardized treatment protocol. Treatment was initiated with a fixed loading phase of up to four monthly intravitreal Ranibizumab injections (identical for all patients), after which patients transitioned to a treat-and-extend regimen in which the injection interval was individually adjusted according to disease activity. The number of intravitreal Ranibizumab injections over the one-year follow-up did not differ significantly between good and poor responders (good: median 9, IQR: 8–9; poor: median 9, IQR: 9–10; *p* = 0.103; range: 8–12 in both groups), indicating an equivalent treatment load across response groups.

Despite the overall functional and anatomical improvements observed in the cohort, the classification into good and poor responders remains clinically meaningful because it is based on validated composite criteria rather than on absolute changes in isolated parameters. Both groups experienced measurable improvements during the one-year follow-up (Table S1 of the Supplementary Materials): the median DBCVA of the overall treated population reached 72 ETDRS letters (IQR: 61–77 letters), and the median CRT with Ranibizumab treatment decreased to 233 µm (IQR: 208–268 µm). Only 20.5% of patients showed residual IRF or SRF at the end of the study, indicating that most eyes demonstrated at least a partial anatomical response to Ranibizumab. Despite these improvements, only 43.2% of the cohort qualified as good responders, defined by complete or near-complete fluid resolution accompanied by a concordant functional and anatomical course, whereas 56.8% were classified as poor/non-responders on the basis of persistent fluid and/or insufficient functional or anatomical improvement.

**Figure 1:**
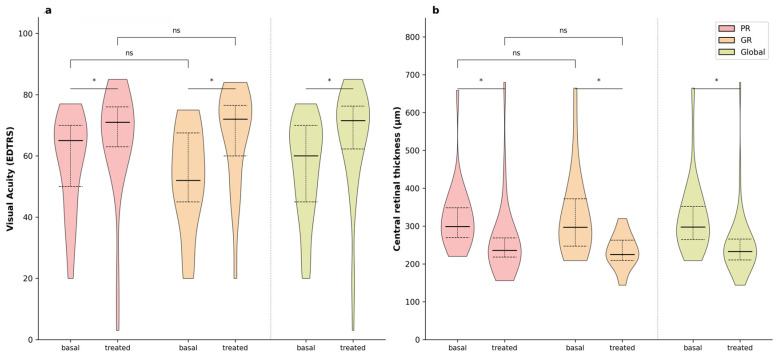
Differences in visual acuity and central retinal thickness between poor responders and good responders before and after treatment of intravitreal Ranibizumab: (**a**) visual acuity (DBCVA); (**b**) central retinal thickness. PR: poor responder; GR: good responder. In each violin plot, the solid horizontal line represents the median and the dashed horizontal lines represent the first and third quartiles. Wilcoxon’s signed-rank test was used to detect differences between parameter levels over time. Mann–Whitney test was used to detect differences between groups. ns: *p*-value > 0.05, * <0.05.

CRT reduction was significant in both groups (*p* < 0.0001), decreasing from 297 µm (IQR: 246–391 µm) to 225 µm (IQR: 207–265 µm) in good responders and from 299 µm (IQR: 268–350 µm) to 236 µm (IQR: 210–272 µm) in poor responders (Figure 1b and Table S1 of the Supplementary Materials). Similarly, although an overall improvement in DBCVA was observed throughout the study period, no significant differences emerged between good and poor responders in DBCVA trajectories (Figure 1a and Table S1 of the Supplementary Materials). The lack of divergence in mean DBCVA and CRT values, therefore, does not invalidate the response classification; rather, it reflects the fact that population averages can mask clinically relevant heterogeneity. Good responders demonstrated concordant anatomical and functional recovery, whereas poor responders showed persistent fluid and/or insufficient anatomical or functional improvement despite treatment.

### 2.2. Biochemical and Inflammatory Parameter Outcomes

Biochemical parameters remained largely comparable between good and poor responders at both the baseline and the final visit. Median levels of urea, uric acid, creatinine, total cholesterol, and triglycerides showed no significant differences between groups and did not identify a distinct metabolic profile associated with treatment response (Table S2, Supplementary Materials). Total cholesterol tended to be slightly lower in poor responders at baseline (178 mg/dL, IQR: 152–212 mg/dL) compared with good responders (192 mg/dL, IQR: 180–227 mg/dL), and this pattern persisted at the final visit (179 mg/dL, IQR: 159–204 mg/dL vs. 184 mg/dL, IQR: 161–211 mg/dL), although the differences were not statistically significant. It should be noted that statin therapy was more frequent among poor responders (60.0%) than good responders (31.6%), although this difference did not reach statistical significance (Fisher’s exact test, *p* = 0.076). The slightly lower cholesterol observed in statin users is an expected pharmacological effect, and, given the confounding by indication inherent to statin prescription, these biochemical patterns should be interpreted as exploratory and cannot be attributed to lipid metabolism or statin exposure independently of the underlying cardiovascular/metabolic conditions that motivate such treatment (Table S2, Supplementary Materials).

**Figure 2:**
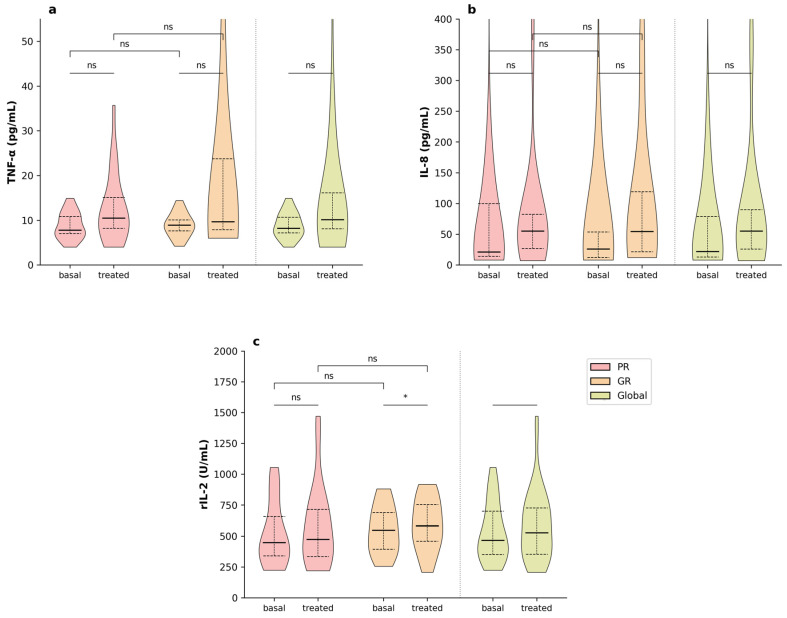
Differences in inflammatory parameters TNF-α, IL-8, and rIL-2 between poor responders and good responders before and after long-term treatment of intravitreal Ranibizumab: (**a**) TNF-α; (**b**) IL-8; (**c**) rIL-2. PR: poor responder; GR: good responder. In each violin plot, the solid horizontal line represents the median and the dashed horizontal lines represent the first and third quartiles. Wilcoxon’s signed-rank test was used to detect differences between parameter levels over time. Mann–Whitney test was used to detect differences between groups (Wilcoxon/Friedman). ns: *p*-value > 0.05, * <0.05.

To determine whether systemic inflammatory activity could better distinguish long-term treatment outcomes, a panel of inflammatory biomarkers was evaluated at baseline and after one year of Ranibizumab treatment to identify possible immune signatures associated with treatment response. Overall, most inflammatory parameters, including white blood cell counts, neutrophils, CRP, ESR, and β2-microglobulin, remained stable over time and did not differ significantly between good and poor responders at either evaluation point (Table S3, Supplementary Materials). TNF-α levels increased modestly in the overall cohort from baseline (8.2 pg/mL, IQR: 7.02–10.8 pg/mL) to the final visit (10.2 pg/mL, IQR: 8.1–16.4 pg/mL), but this rise did not reach statistical significance and was similar across response groups (Figure 2a).

Among the cytokines analyzed, soluble IL-2 receptor (IL-2R) and IL-8 were the only biomarkers showing significant intra-individual variation over the study period. Of these two parameters, rIL-2 increased significantly from baseline to the final visit in the overall cohort (466 to 527 U/mL; *p* value = 0.023), and this change remained statistically significant in good responders (*p*-value = 0.006). Although rIL-2 values did not differ significantly between groups at baseline or at one-year, good responders consistently presented higher median levels at both time points (baseline: 547 vs. 448 U/mL; final: 582 vs. 472 U/mL) (Figure 2c). This trend suggests the presence of a more pronounced immunomodulatory profile among good responders, even if the differences did not reach statistical significance. IL-8 concentrations also increased over time in the global analysis (from 22 to 55 pg/mL; Friedman’s *p* value = 0.045), but no meaningful separation was observed between response groups at baseline or at long-term follow-up (Figure 2b). Other cytokines, including IL-6, IL-1β, IL-5, and IL-10, remained unchanged throughout the study period, with no differences between responders and poor responders.

### 2.3. miRNA Expression in the Long-Term Analysis

**Figure 3:**
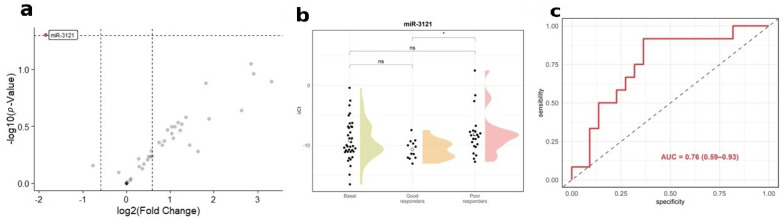
(**a**) Volcano plot depicting differential miRNA expression profile of poor responders compared with good responders in the long-term response after applying the requisite miRNA sequencing quality assurance criterion. The log2 (fold change) between poor responders and good responders is shown on the x-axis. The y-axis represents the log10 (q-value) by *t*-test method. Vertical dashed lines mark the fold-change threshold. The red point highlights miR-3121 as the significantly dysregulated miRNA; gray points represent other miRNAs that did not reach statistical significance. (**b**) Half-violin plus dot-plot showing miR-3121 expression levels (ΔCt) across clinical response groups: basal samples (yellow), good responders at final visit (orange), and poor responders at final visit (pink/red). Individual data points are shown as gray dots. Horizontal lines indicate median (solid) and quartiles (dashed). (**c**) Receiver operating characteristic (ROC) curve evaluating miR-3121 (ΔCt) performance in distinguishing good from poor responders at final visit. The solid red line represents the classifier performance; the diagonal dashed gray line represents chance performance (AUC = 0.5). The model achieved an AUC of 0.76 (95% CI: 0.59–0.93), indicating moderate discriminative ability. ns: *p*-value > 0.05; *: *p*-value < 0.05.

An analysis of miRNA expression was performed in 34 patients (10 good responders and 24 poor responders) to determine whether systemic miRNA signatures could differentiate long-term response profiles to Ranibizumab. A total of 35 miRNAs showed differential expression between poor responders and good responders in the cohort after applying a log_2_FC|≥0.58| threshold: three downregulated and 32 upregulated in poor responders (Table S4, Supplementary Materials). Among these, only one miRNA reached statistical significance after fold change and *p*-value filtering—miR-3121—which was significantly downregulated in good responders compared with poor responders (fold change = 1.852; *p* = 0.0496) (Figure 3a). This differential expression is shown in the volcano and violin plots (Figure 3b) and represents the only miRNA with discriminative potential in the long-term analysis. To evaluate the potential of miR-3121 as a biomarker of treatment response, receiver operating characteristic (ROC) curves were generated, and the area under the curve (AUC) was calculated. The model achieved an AUC of 0.76 (95% CI: 0.59–0.93), indicating a moderate discriminative ability to distinguish between poor and good responders (Figure 3c).

No other miRNA demonstrated statistically significant differences between good and poor responders, although several candidates exhibited directional trends in pathways relevant to angiogenesis, inflammation, and lipid regulation.

## 3. Discussion

The present study provides an integrated evaluation of clinical, biochemical, inflammatory, and miRNA profiles in patients with nAMD treated with Ranibizumab over a one-year period. Although both good and poor responders experienced anatomical and functional improvements consistently with the expected therapeutic effect of anti-VEGF therapy, the classification into response groups revealed distinct systemic features that may contribute to long-term outcomes. Our findings suggest that subtle differences in immune regulation and miRNA expression may play a relevant role in shaping treatment response, while metabolic parameters remained largely comparable between groups.

As observed in the clinical parameters analyzed, despite more than half of the cohort being classified as poor responders, both groups experienced significant reductions in CRT and a general stabilization or improvement in vision. This apparent inconsistency highlights the limitations of relying solely on mean DBCVA or CRT values when evaluating treatment outcomes. Composite criteria that include anatomical resolution, percentage reductions in retinal thickness, and functional thresholds more accurately capture individual therapeutic responses and better reflect the complexity of neovascular AMD. The high proportion of poor responders in the present cohort, therefore, represents the strictness of these criteria rather than insufficient treatment efficacy; fortunately, this limitation is progressively mitigated with newer and more potent anti-VEGF agents, such as Faricimab or high-dose Aflibercept, which achieve deeper and more sustained VEGF suppression and have demonstrated improved outcomes in patients with a suboptimal response to first-generation antibodies [3,4].

Upon analysis of the patients’ metabolic parameters, including cholesterol, triglycerides, creatinine, urea, uric acid, and CKD-EPI, no differences were found between response groups after one year of treatment. We observed a non-significant trend toward more frequent statin use among poor responders (60.0% vs. 31.6%; Fisher’s exact test, *p* = 0.076), a pattern also noted in the short-term response analysis of this cohort [5]. This observation must be interpreted with caution and as hypothesis-generating only, as it is subject to confounding by indication: statins are prescribed precisely to patients with dyslipidemia and elevated cardiovascular/metabolic risk, so statin use serves as a proxy for that underlying condition. With the present sample size (*n* = 44), formal multivariable adjustment for comorbidity was not feasible, and cardiovascular/metabolic conditions such as hypertension and diabetes were not systematically recorded; consequently, the effect of statins themselves cannot be distinguished from that of the underlying vascular/metabolic disease. While statins are known to exert dual, dose-dependent effects on angiogenesis and pleiotropic actions on endothelial and inflammatory signaling [6,7] that could, in principle, modulate anti-VEGF responsiveness, our data do not allow any causal inference, and this question would require adequately powered, prospectively designed studies recording full cardiovascular/metabolic comorbidity.

Cytokines play a central role in the pathophysiology of AMD, contributing directly to cellular damage or indirectly through mechanisms like increasing blood–retinal barrier permeability and upregulating VEGF expression, which promotes neovascularization [8]. IL-2 is a cytokine involved in the control of immune responses, and its dysregulation plays a role in AMD by contributing to inflammation and fibrosis. Studies suggest that in the context of AMD, IL-2 promotes the migration of retinal pigment epithelial (RPE) cells and the excessive production of extracellular matrix (ECM), which can lead to macular fibrosis [9]. When receptor IL-2 levels increase, they can capture free IL-2, limiting its ability to bind to its receptors on T cells; this can lead to transient immunosuppression, impairing the immune response that normally depends on IL-2. Although statistical significance was not found between response groups, in our case, both baseline and final values of rIL-2 were higher in the responder group compared to the non-responder group. Therefore, the immunosuppressive effect could favor a better response to anti-VEGF treatment and support the theory of inflammatory dysregulation in AMD.

Elevated levels of TNF-α, IL-1β, and IL-8 are correlated with increased vascular permeability and disease progression; in addition, elevated levels of IL-8 in aqueous humor were associated with poor outcomes from Bevacizumab treatment at week 12 [10], although opposing results have also been reported by other studies in aqueous humor [11]. We only found one study that related treatment response to serum levels of IL-8. In this study, those patients who had gone through longer treatment periods with anti-VEGF before sampling had significantly higher levels of IL-8 and VEGF-A than those that received treatment in less than two months of sample extraction [12]. In our case, IL-8 serum levels increased over time in all patients, and although higher levels were found in the good responder group, no statistical differences were found between groups. The cellular origin of the cytokine changes observed here cannot be determined from soluble plasma measurements. Based on the established literature, soluble IL-2R is shed mainly by activated T lymphocytes and reflects T-cell/immune activation, whereas IL-8 derives from monocytes/macrophages, peripheral blood mononuclear cells, and activated endothelial and RPE cells, with elevated PBMC- and serum-derived IL-8 previously reported in nAMD. These attributions are interpretive; cell-type-resolved profiling (e.g., flow cytometry or single-cell approaches) would be required to confirm the cellular sources, and this is a relevant direction for future work.

MiRNAs are key post-transcriptional regulators involved in inflammation, angiogenesis, and oxidative stress, all of which contribute to AMD pathogenesis. Among the panel analyzed, miR-3121 was the only miRNA showing significant differential expression between response groups; this miRNA has been implicated in immune regulation and vascular processes in other diseases, although its specific role remains poorly defined [13,14,15]. We selected this miRNA from a study with a cohort of 300 patients (150 with neovascular AMD and 150 with dry AMD) and 200 controls. In this study, miR-3121 blood levels were higher in patients with AMD than in healthy controls; additionally, if comparisons are made within AMD classification, mir-3121 is three times upregulated in dry AMD than in wet AMD [16]. No other studies have been found that relate this biomarker to AMD, nor to its possible association with treatment response. In our case, differences are observed between patients who obtain a complete response vs. those who obtain a partial response, although the significance level is very tight (*p* = 0.0496). As this biomarker seems to be upregulated by the disease itself, it is possible that this miR-3121 being downregulated in good responders could be considered a possible biomarker of treatment response.

miR-3121 is a poorly characterized microRNA with no previously described role in retinal disease. Its best-validated target is Rap1GAP, a direct repression target of miR-3121-3p [14], and it has additionally been associated with the regulation of immune- and vascular-related genes [14,17]. Mechanistically, Rap1GAP inactivates the small GTPase Rap1; reduced miR-3121 would, therefore, be expected to de-repress Rap1GAP and attenuate Rap1 activity. As Rap1 is a positive regulator of VEGFR2 signaling, VEGF-induced angiogenesis, and endothelial barrier function [18], this Rap1GAP–Rap1–VEGFR2 axis offers a plausible route by which miR-3121 could influence the pathway targeted by Ranibizumab, consistent with the direction of our findings. This proposed mechanism is hypothesis-generating: it is extrapolated from non-ocular contexts, the Rap1 axis is context-dependent, and it was not experimentally validated in retinal cells or in the present study.

Studies that relate miRNAs to treatment response are scarce—we found only two. One analyzed a short-term response [18], and the other, a long-term response with a pro re nata treatment regime [18]; the first one identified a panel of 18 miRNAs that predicted the short-term response to Ranibizumab [18], while the other identified miR-195-5p and miR-185-5p in tears and blood with long-term treatment outcomes [19]. None of these miRNAs overlapped with ours, likely reflecting methodological differences, cohort heterogeneity, and the biological diversity of AMD.

Although this study focuses on the response to Ranibizumab, several of the analyzed miRNAs are known modulators of lipid metabolism, inflammatory processes, or angiogenesis. miR-125b overexpression can protect RPE cells from oxidative damage by regulating the Nrf2/HIF-1α signaling pathway [20]. miR-106b acts as a potent inhibitor of abnormal blood vessel formation, and reducing its levels in the retina triggers vascular growth by inducing the production of pro-angiogenic factors [21]. Lower expression of let-7b might indicate resistance to Ranibizumab treatment [22]. miR-155 is downregulated in AMD patients. Upregulation of miR-155 increases the expression of IFN-β, which controls the expression of SOCS-1 and IL-10, two important anti-inflammatory mediators, thus controlling the immune response [23].

Overall, the profile observed in non-responders suggests impaired lipid and inflammatory homeostasis, both relevant to angiogenesis and anti-VEGF efficacy. Therefore, patients who do not respond to treatment exhibit an altered miRNA–lipid–inflammatory profile. The non-significant trend toward higher statin use among poor responders adds a further layer of complexity that, as noted above, cannot be disentangled from confounding by indication. Statins are known to alter miRNA expression, extending their effects beyond simple cholesterol reduction to complex cellular and epigenetic regulation [24,25], possibly and indirectly affecting the efficacy of Ranibizumab; therefore, this potential interaction requires further investigation.

Future research should aim to validate these findings in larger cohorts regarding the predictive value of miRNA signatures and inflammatory markers. Standardization of miRNA detection and normalization methods is also essential, as current protocols vary widely across studies, even in those investigating miRNAs as diagnostic biomarkers [14,26], hindering direct comparisons and clinical translation. Expanding the miRNA panel to include broader transcriptomic or multi-omic approaches may reveal additional regulatory networks involved in the anti-VEGF response. In parallel with the identification of predictive biomarkers, emerging therapeutic strategies—including sustained-release and nanocarrier-based delivery systems designed to enhance retinal bioavailability and reduce the burden of repeated intravitreal injections—may broaden the management options for nAMD and warrant integration with response-stratification approaches in future work [27,28].

This study has several strengths, including its real-world design, prospective follow-up, and the integration of clinical, biochemical, inflammatory, and molecular parameters, offering a comprehensive view of systemic contributors to Ranibizumab response. However, the strict inclusion criteria reduced the sample size, which may have limited statistical power, so bigger studies are necessary to validate the results. We centered this miRNA analysis on the long-term response to Ranibizumab treatment due to the observed loss of treatment response over time in clinical practice, yet short-term dynamics were not explored. Lastly, our study focused solely on a selected set of miRNAs, and intraocular biomarkers were not measured, potentially overlooking the involvement of other miRNAs in Ranibizumab treatment response.

## 4. Materials and Methods

### 4.1. Patient Selection Criteria

In this prospective observational, one-year study of patients with untreated nAMD, who were eligible for initiation of Ranibizumab based on clinical practice, patients were recruited and examined by ophthalmologists at the University Hospital of Santiago de Compostela. After an initial clinical examination (baseline measurements), patients received up to four monthly intravitreal injections of Ranibizumab (Lucentis©, Novartis, Basel, Switzerland), followed by a treat-and-extend (T&E) regimen. Anatomical changes were evaluated at baseline and at one year after treatment initiation using ophthalmoscopy, fluorescein angiography, optical coherence tomography (OCT), and OCT angiography. The study was conducted in accordance with the Declaration of Principles of Helsinki, with Ethical approval obtained from the local Institutional Review Board and the Ethics Committee of the Autonomous Community of Galicia (2017/614).

Inclusion criteria were defined as: neovascularization secondary to AMD; age over 55 years; and a baseline Early Treatment Diabetic Retinopathy Study (ETDRS) Distance Best Corrected Visual Acuity (DBCVA) score of more than 25 letters. Exclusion criteria included: other retinal diseases unrelated to nAMD; prior ophthalmic surgery other than cataract surgery; and any signs of intraocular inflammation. No subject had previously received anti-VEGF therapy for nAMD.

### 4.2. Classification of Clinical Response

The clinical response to Ranibizumab was assessed 12 months after treatment initiation. Treatment response was classified following the framework of Amoaku et al. [1], which evaluates morphological and functional responses as complementary dimensions. The resolution of IRF and/or SRF on OCT was the primary morphological criterion, assessed jointly with the functional (DBCVA) and anatomical (CRT) course over the follow-up. Optimal responders were patients showing complete or near-complete resolution of IRF and/or SRF together with concordant functional and anatomical improvements. Patients with persistent or new IRF/SRF, or in whom fluid resolution was accompanied by functional decline or insufficient anatomical improvement, were classified as poor responders. CRT reduction and ETDRS letter change are reported as descriptive parameters of response (Table 1 and Table S1) rather than as fixed thresholds.

### 4.3. Data Collection

Demographic characteristics, Body Mass Index (BMI), smoking history, comorbidities, and concomitant treatments were recorded at the baseline visit. Blood tests were performed at the initial visit and at one year after the initiation of Ranibizumab treatment. These tests included basic biochemical parameters, a complete blood count, miRNAs, and inflammatory biomarkers.

### 4.4. Cytokine Determination

Patient plasma samples were collected at all visits, isolated from venous blood using an EDTA tube (4 mL), and stored frozen at −80 °C until analysis. A selection of inflammatory biomarkers was carried out through the development of a bibliographic search based on the scientific evidence so far from articles related to AMD. The following cytokines were measured using an IMMULITE analyzer (Siemens Healthcare GmbH, Erlangen, Germany): IL-1b, IL-2R, IL-5, IL-6, IL-8, IL-10, and TNF-α. IMMULITE 1000 was employed for IL2-R, IL-8, IL-10, and TNF, and INMULITE 2000 for IL-6 and IL-2R. Both devices used reagents and instructions provided by the manufacturer.

### 4.5. miRNA Determination

Peripheral venous blood was collected at the baseline into two 4 mL EDTA tubes. Leukocytes were separated within 24 h. Briefly, 5 mL of whole blood was centrifuged at 2280× *g* for 15 min at room temperature. The leukocyte layer was recovered, washed twice with saline, resuspended in 500 µL of saline, and stored at −80 °C until RNA isolation.

A targeted panel of miRNA candidates was assembled based on a literature review of the scientific evidence and studies related to AMD and pharmacogenetics, resulting in the evaluation of 57 publications. From these, 37 miRNA biomarkers with the strongest supporting evidence were selected, encompassing biomarkers associated with treatment response as well as AMD susceptibility and pathogenesis.

For the extraction of miRNAs, the mirVana extraction method was chosen. Once the genetic material was extracted, retrotranscription, preamplification, and normalization were performed to obtain complementary DNA (cDNA). For the panel design, genotyping was performed using a custom OpenArray™ platform on the QuantStudio™ 12K Flex Real-Time PCR System (Life Technologies, Carlsbad, CA, USA), based on TaqMan^®^ technology. Data analysis was performed using QuantStudio™ 12K Flex Real-Time PCR Software (version 1.2) and TaqMan^®^ Genotyper Software (version 1.3), which enables biomarker-level cluster analysis.

### 4.6. Statistical Analysis

Data were analyzed with SPSS Statistics v20^®^ (IBM SPSS Statistics) and R version 4.6.0 for Windows^®^ using RStudio 2026.05.1. Wilcoxon’s rank sum test and Friedman’s test were used to detect differences between biomarker levels over time. Differences between good responder versus poor responder groups were explored using statistical tests; the non-parametric Mann–Whitney test in the case of quantitative variables, and the chi-square test or Fisher’s exact test in the case of categorical variables.

## 5. Conclusions

The findings of this study indicate that the one-year response to Ranibizumab in neovascular AMD may be associated with the interplay between lipid metabolism, inflammation, and miRNA regulation. A non-significant trend toward higher statin use among poor responders was observed; however, owing to confounding by indication and the limited sample size, this cannot be interpreted as evidence that lipid-lowering therapy influences anti-VEGF efficacy, and it should be regarded as an exploratory observation requiring dedicated investigation.

MiRNAs participate in fundamental biological processes of AMD. In this context, the differences in miR-3121 expression between good and poor responders suggest its potential utility as a therapeutic response biomarker. There are no previous studies that directly link this miRNA to AMD therapeutic response, which reinforces interest in it as a candidate for future research. Validation in larger cohorts is required to confirm the clinical value of miR-3121 and other potential miRNAs as markers of treatment response.

Taken together, these results support the need for a more personalized approach to the treatment of neovascular AMD, integrating inflammatory, metabolic, and molecular profiles to optimize the response to anti-VEGF therapies.

## Figures and Tables

**Table 1:** Baseline demographics, clinical characteristics, and concomitant medications of the study population according to long-term treatment response (*p* values derived from Student’s *t*-test).

		Long-Term Response		
Variables	Total (*n* = 44)	Poor Responders (*N* = 25)	Good Responders (*N* = 19)	*p*-Value
Sex (women) N %	26, 59.1	16, 64.0	10, 52,6	0.542
Age (years) Median, IQR	80, (74–86)	80, (74–86)	78, (75–87)	0.635
**Co-morbidities**				
Obesity (>30 kg/m^2^)	17, 38.6	9, 36.0	8, 42.1	0.760
Smoker	14, 31.8	8, 32.0	6, 31.6	0.976
**Concomitant medication**				
Anti-inflammatory	13, 29.5	7, 28.0	6, 31.6	0.797
Anticoagulant	9, 20.5	5, 20.0	4, 21.1	0.932
Statin	21, 47.7	15, 60.0	6, 31.6	0.076

## Data Availability

The original contributions presented in this study are included in the article and Supplementary Materials. Further inquiries can be directed to the corresponding authors.

## Supplementary Materials

The following supporting information can be downloaded at: https://www.mdpi.com/article/10.3390/ph19060955/s1, Table S1: Clinical parameters on long-term response analysis; Table S2: Biochemical parameters analysed by treatment response; Table S3: Inflammatory parameters results analysed by treatment response; Table S4: MiRNAs expressed in the long-term analysis.

## Abbreviations

The following abbreviations are used in this manuscript:

AMD	Age-related macular degeneration
CRP	C-reactive protein
CRT	Central retinal thickness
ETDRS	Early Treatment Diabetic Retinopathy Study
IRF	Intraretinal fluid
nAMD	Neovascular age-related macular degeneration
OCT	Optical coherence tomography
OR	Odds Ratio
PDT	Photodynamic therapy
PRN	Pro re nata
SRF	Subretinal fluid
T&E	Treat and extend
VA	Visual acuity
VEGF	Vascular endothelial growth factor
