# Supplementary material for: Systemic Immune and miRNA Signatures Associated with Long-Term Ranibizumab Response in Neovascular Age-Related Macular Degeneration
Source: Pharmaceuticals (Basel). 2026 Jun 19;19(6):955. doi: 10.3390/ph19060955 (PMC13304664; doi:10.3390/ph19060955)
Supplement: Supplementary file 1 [file pharmaceuticals-19-00955-s001.zip › Table S1.pdf]

Table S1. Clinical parameters on long-term response analysis

	Total (n=44)	Poor Responders (n= 25)	Good responders (n= 19)	p-value
**Response parameters**				
AV (EDTRS)				
Basal	60, (45-70)	65, (46-70)	52, (45-70)	0.264
Treated	72, (61-77)	71, (62-76)	72, (55-77)	0.687
Wilcoxon	0.001	0.037	0.001	
CRT (μm)				
Basal	298, (260-354)	299, (268-350)	297, (246-391)	0.906
Treated	233, (208- 268)	236, (210-272)	225, (207-265)	0.636
Wilcoxon	0.001	0.002	0.001	
IRF (%)				
Basal	27, 61.4	13,52.0	14, 73.7	0.213
Treated	9, 20.5	8, 32.0	1, 5.3	0.029
SRF, (%)				
Basal	33, 75.0	20, 80.0	13, 68.4	0.347
Treated	9, 20.5	9, 36.0	0, 0.0	0.014

Parameters: Median, IQR: interquartile range. CRT: central retinal thickness, ETDRS: Early Treatment Diabetic Retinopathy Study, IRF: Intraretinal fluid, SRF: subretinal fluid, RPE: retinal pigment epithelium, VA: Visual Acuity
