# Supplementary material for: Systemic Immune and miRNA Signatures Associated with Long-Term Ranibizumab Response in Neovascular Age-Related Macular Degeneration
Source: Pharmaceuticals (Basel). 2026 Jun 19;19(6):955. doi: 10.3390/ph19060955 (PMC13304664; doi:10.3390/ph19060955)
Supplement: Supplementary file 1 [file pharmaceuticals-19-00955-s001.zip › Table S2.pdf]

**Table S2. Biochemical parameters analysed by treatment response**

| | **Total (N=44)** | **Poor responders (N= 25)** | **Good responders (N= 19)** | **p-value** |
|---|---|---|---|---|
| **Urea** (mg/dL) | | | | |
| Basal | 44, (34-54) | 42, (33-53) | 49, (35-68) | 0.139 |
| Treated | 49, (39-62) | 44, (38-60) | 55, (45-69) | 0.109 |
| Wilcoxon | 0.027 | 0.025 | 0.365 | |
| **Uric Acid (**mg/dL) | | | | |
| Basal | 5.1, (3.8-6.0) | 5.1, (3.9-6.4) | 5.0, (3.6-5.9) | 0.522 |
| Treated | 5.5, (3.8-6.5) | 5.5, (4.2-6.8) | 4.9, (3.6-6.5) | 0.226 |
| Wilcoxon | 0.204 | 0.186 | 0.660 | |
| **Creatinine** (mg/dL) | | | | |
| Basal | 0.91, (0.72-1.07) | 0.91, (0.70-1.02) | 0.90, (0.72-1.22) | 0.571 |
| Treated | 0.96, (0.76-1.20) | 0.97, (0.74-1.11) | 0.90, (0.78-1.24) | 0.924 |
| Wilcoxon | 0.015 | 0.005 | 0.602 | |
| GFR | | | | |
| Basal | 69.8, (57.6-83.7) | 70.3, (57.5-84.2) | 68.0, (53.8-81.4) | 0.649 |
| Treated | 62.9, (51.1-79.4) | 62.9, (52.3-80.2) | 63.1, (46.0-80.4) | 0.749 |
| Wilcoxon | 0.004 | 0.002 | 0.435 | |
| **Cholesterol** | | | | |
| Basal | 188, (163-216) | 178, (152-212) | 192, (180-227) | 0.087 |
| Treated | 182, (160-210) | 179, (159-204) | 184, (161-211) | 0.420 |
| Wilcoxon | 0.223 | 0.667 | 0.214 | |
| **Triglycerides** | | | | |
| Basal | 105, (71-145) | 106, (70-154) | 100, (54-117) | 0.658 |
| Treated | 104, (69-140) | 111, (68-144) | 95, (73-137) | 0.906 |
| Wilcoxon | 0.261 | 0.206 | 0.663 | |

Parameters: median, IQR. Reference range: Urea: 12-44 mg/dL; Uric Acid (UA): 3,5-7,2 mg/dL, creatinine: 0,63-1,13 mg/dL;
