# Supplementary material for: Systemic Immune and miRNA Signatures Associated with Long-Term Ranibizumab Response in Neovascular Age-Related Macular Degeneration
Source: Pharmaceuticals (Basel). 2026 Jun 19;19(6):955. doi: 10.3390/ph19060955 (PMC13304664; doi:10.3390/ph19060955)
Supplement: Supplementary file 1 [file pharmaceuticals-19-00955-s001.zip › Table S3.pdf]

Table S3. Inflammatory parameters results analysed by treatment response

	Total (N=44)	Poor responders (N= 25)	Good responders (N= 19)	p-value
WBC **(x10^3)**				
Basal	6.92, (5.44-7.70)	6.96, (5.63-8.00)	6.70, (5.35-7.91)	0.749
Treated	6.18, (5.04-7.37)	6.17, (5.42-7.80)	6.33, (4.82-7.39)	0.868
Wilcoxon	0.340	0.563	0.486	
neutrophils (x103)				
Basal	4.48, (3.51-5.4)	4.48, (3.46-5.31)	4.28, (3.58-6.09)	0.912
Treated	3.93, (3.09-4.98)	3.80, (3.10-5.46)	4.27, (3.08-4.93)	0.924
Wilcoxon	0.413	0.775	0.338	
CRP **(mg/dL)**				
Basal	0.190, (0.120-0.410)	0.213, (0.120-0.615)	0.155, (0.095-0.310)	0.188
Treated	0.230, (0.115-0.375)	0.270, (0.180-0.440)	0.155, (0.110-0.338)	0.176
Wilcoxon VF vs V1	0.607	0.795	0.224	
ERS **(mm)**				
Basal	22.0, (10.0-38.0)	19.0, (9.0-28.0)	22.5, (13.8-42.5)	0.475
Treated	27.0, (8.0-40.0)	15.0, (6.5-37.0)	29.0, (17.5-41.0)	0.118
Wilcoxon VF vs V1	0.902	0.242	0.205	
B2M **(mg/L)**				
Basal	2.57, (1.99-3.35)	2.48, (1.98-3.20)	2.70, (2.00-3.71)	0.537
Treated	2.81, (2.05-3.68)	2.83, (2.05-3.94)	2.76, (2.12-3.38)	0.844
Wilcoxon VF vs V1	0.346	0.360	0.605	
TNF **(pg/mL)**				
Basal	8.2, (7.02-10.8)	7.8, (6.68-11.1)	8.9, (7.2-10.6)	0.694
Treated	10.2, (8.1-16.4)	10.5, (8.2-16.1)	9.6, (7.5-28.4)	0.828
Wilcoxon VF vs V1	0.082	0.204	0.246	
rIL-2 **(U/mL)**				
Basal	466, (345-711)	448, (329-711)	547, (384-711)	0.429
Treated	527, (351-736)	472, (324-723)	582, (443-773)	0.399
Wilcoxon VF vs V1	0.023	0.601	0.006	
IL-6 **(pg/mL)**				
Basal	6.1, (4.2-8.2)	7.0, (4.4-8.2)	5.4, (4.0-7.4)	0.416
Treated	5.3, (3.3-8.6)	6.1, (4.8-9.3)	4.8, (2.6-7.4)	0.092
Wilcoxon VF vs V1	0.586	0.922	0.435	
IL-8 **(pg/mL)**				
Basal	22, (13-100)	21, (14-118)	26, (12-81)	0.786
Treated	55, (24-106)	55, (26-90)	54, (20-142)	0.969
Wilcoxon VF vs V1	0.112	0.260	0.309	

Parameters: median, IQR. B2M: Beta-2-Microglobulin, CRP: C-reactive protein, ESR: erythrocyte sedimentation rate, TNF: tumor necrosis factor; WBC: white blood count.
Reference range: B2M: 1,09-2,53 mg/L; CRP: 0-0,5 mg/dL: ESR: 0-20 mm; rIL-2: 158-623 U/mL; IL-6: 0-5 pg/mL; IL-8: 0-62 pg/mL; TNF: 0-8,1 pg/mL.
