# Supplementary material for: Systemic Immune and miRNA Signatures Associated with Long-Term Ranibizumab Response in Neovascular Age-Related Macular Degeneration
Source: Pharmaceuticals (Basel). 2026 Jun 19;19(6):955. doi: 10.3390/ph19060955 (PMC13304664; doi:10.3390/ph19060955)
Supplement: Supplementary file 1 [file pharmaceuticals-19-00955-s001.zip › Table S4.pdf]

Table S4. MiRNAs expressed in the long-term analysis.

downregulated			up regulated					
**miRNA name**	**Fold**	**p-value**	**miRNA name**	**Fold**	**p-value**	**miRNA name**	**Fold**	**p-value**
miR-152-3p	0,182	0.802	let-7a-5p	0,592	0,574	miR-27a	0,273	0,610
miR-31	0,768	0.701	let-7b-5p	0,483	0,620	miR-29b	1,811	0,131
miR-3121	1,852	0.049	let-7c-5p	1,071	0,400	miR-301-5p	1,437	0,46
			let-7d-5p	0,295	0,711	miR-301a-3p	0,559	0,519
			miR-106a-5p	1,179	0,291	miR-323-3p	0,358	0,740
			miR-106b-5p	0,018	0,973	miR-342-3p	1,354	0,263
			miR-125b-5p	2,632	0,230	miR-410	0,391	0,679
			miR-146a-5p	0,098	0,912	miR-424-5p	2,904	0,108
			miR-150	0,082	0,944	miR-4258	1,224	0,344
			miR-155	1,257	0,300	miR-574-3p	1,029	0,365
			miR-17-5p	0,941	0,338	miR-660-5p	0,553	0,585
			miR-192-5p	0,504	0,583	miR-661	2,844	0,089
			miR-21-5p	0,859	0,426	miR-885-5p	0,818	0,460
			miR-221-5p	1,107	0,319	miR-889	3,310	0,127
			miR-23a-3p	1,636	0,5263	miR-9-5p	1,887	0,272
			miR-24-3p	0,745	0,437	mir-191-5p	1,039	0,316
